# Supplementary material for: Identification of photoperiod-induced specific miRNAs in the adrenal glands of Sunite sheep (Ovis aries)
Source: Front Vet Sci. 2022 Jul 22;9:888207. doi: 10.3389/fvets.2022.888207 (PMC9354845; doi:10.3389/fvets.2022.888207)
Supplement: Supplementary file 1 [file Data_Sheet_1.ZIP › Supplementary file/miRDeep2 file.zip!/miRDeep2 file/Know_Structure/SP42A1/oar-mir-3958.pdf]

miRBase precursor : oar-mir-3958
Total read count : 10806
oar-miR-3958-5p read count : 10
oar-miR-3958-3p read count : 10796
remaining reads : 0

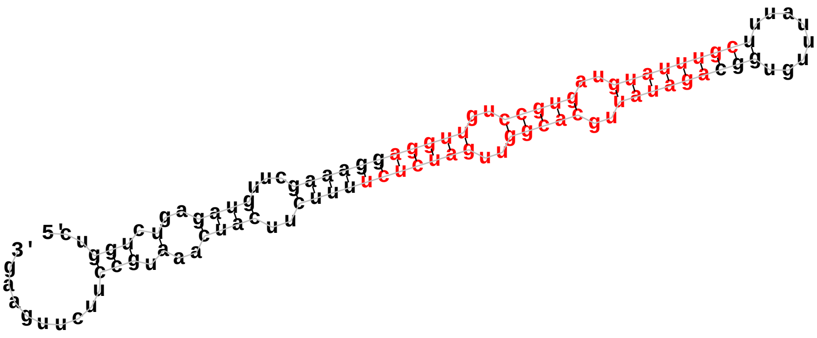

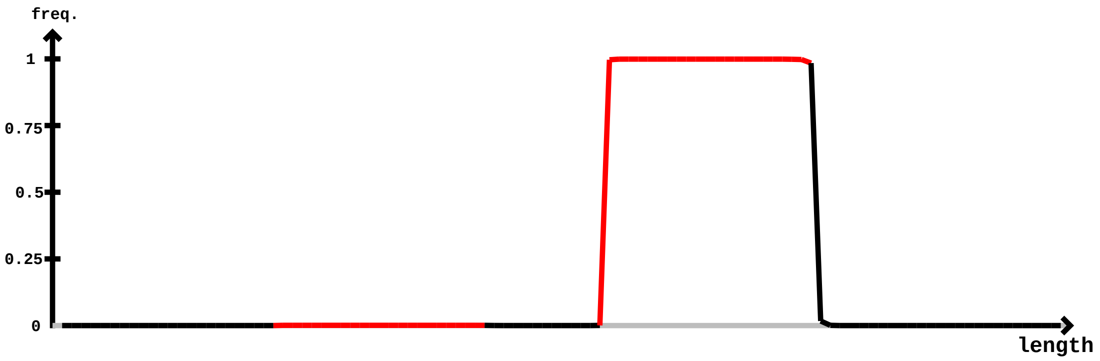


```
                                                       oar-miR-3958-3p
                      oar-miR-3958-5p
5'- cuggucugagauguucgaaaggagguuguccgugauguauuugcuuuauuuguggcagauauugcacgguugaucucuuuucuucaucaaaugccuucuugaag   -3'   exp
    ..(((.(..((((...(((((((((((..(((((..(((((((((........)))))))))..)))))..)))))))))))..))))..).))).........   reads   mm   sample
    ......................agguuguccgugauguauuug.............................................................   1       0    seq
    ......................agguuguccgugauguauuugc............................................................   7       0    seq
    ......................agguuguccgugauguauuugcu...........................................................   2       0    seq
    .......................................................cagauauugcacgguugaucucu..........................   6       0    seq
    ........................................................agauauugcacgguugauc.............................   6       0    seq
    ........................................................agauauugcacgguugaucu............................   10      0    seq
    ........................................................agauauugcacgguugaucuc...........................   144     0    seq
    ........................................................agauauugcacgguugaucucu..........................   10419   0    seq
    ........................................................agauauugcacgguugaucucuu.........................   175     0    seq
    ........................................................agauauugcacgguugaucucuuu........................   10      0    seq
    .........................................................gauauugcacgguugaucuc...........................   1       0    seq
    .........................................................gauauugcacgguugaucucu..........................   24      0    seq
    ..........................................................auauugcacgguugaucucu..........................   1       0    seq
```
